# Recent Progress in Multifunctional Stimuli-Responsive Combinational Drug Delivery Systems for the Treatment of Biofilm-Forming Bacterial Infections

**DOI:** 10.3390/pharmaceutics16080976

**Published:** 2024-07-24

**Authors:** Davoodbasha MubarakAli, Kandasamy Saravanakumar, Archchana Ganeshalingam, Sugavaneswaran Siva Santosh, Shanali De Silva, Jung Up Park, Chang-Min Lee, Su-Hyeon Cho, Song-Rae Kim, Namki Cho, Gobika Thiripuranathar, SeonJu Park

**Affiliations:** 1 School of Life Sciences, B.S. Abdur Rahman Crescent Institute of Science and Technology, Chennai 600048, Tamil Nadu, India; mubinano@gmail.com; 2 Research Institute of Pharmaceutical Sciences, College of Pharmacy, Chonnam National University, Gwangju 61186, Republic of Korea; saravana732@gmail.com (K.S.); cnamki@jnu.ac.kr (N.C.); 3 Center of Molecular Medicine and Diagnostics (COMManD), Department of Biochemistry, Saveetha Dental College & Hospitals, Saveetha Institute of Medical and Technical Sciences, Saveetha University, Chennai 600077, Tamil Nadu, India; 4 College of Chemical Sciences, Institute of Chemistry Ceylon, Welikada, Rajagiriya 10107, Sri Lanka; archchana@ichemc.edu.lk (A.G.); shanali@ichemc.edu.lk (S.D.S.); 5 Technical University of Munich, Campus Straubing, 94315 Straubing, Germany; siva10santosh@gmail.com; 6 Division of Practical Application, Honam National Institute of Biological Resources, 99, Gohadoan-gil, Mokpo-si 58762, Republic of Korea; pju2560@hnibr.re.kr; 7 Department of Veterinary Internal Medicine, College of Veterinary Medicine and BK21 FOUR Program, Chonnam National University, Gwangju 61186, Republic of Korea; cmlee1122@jnu.ac.kr; 8 Gwangju Center, Korea Basic Science Institute (KBSI), Gwangju 61751, Republic of Korea; chosh93@kbsi.re.kr; 9 Metropolitan Seoul Center, Korea Basic Science Institute (KBSI), Seoul 03759, Republic of Korea; ksr87@kbsi.re.kr

**Keywords:** bacterial infection, stimuli-responsive, drug delivery systems, combination therapy

## Abstract

Drug-resistant infectious diseases pose a substantial challenge and threat to medical regimens. While adaptive laboratory evolution provides foresight for encountering such situations, it has inherent limitations. Novel drug delivery systems (DDSs) have garnered attention for overcoming these hurdles. Multi-stimuli responsive DDSs are particularly effective due to their reduced background leakage and targeted drug delivery to specific host sites for pathogen elimination. Bacterial infections create an acidic state in the microenvironment (pH: 5.0–5.5), which differs from normal physiological conditions (pH: 7.4). Infected areas are characterized by the overexpression of hyaluronidase, gelatinase, phospholipase, and other virulence factors. Consequently, several effective stimuli-responsive DDSs have been developed to target bacterial pathogens. Additionally, biofilms, structured communities of bacteria encased in a self-produced polymeric matrix, pose a significant challenge by conferring resistance to conventional antimicrobial treatments. Recent advancements in nano-drug delivery systems (nDDSs) show promise in enhancing antimicrobial efficacy by improving drug absorption and targeting within the biofilm matrix. nDDSs can deliver antimicrobials directly to the biofilm, facilitating more effective eradication of these resilient bacterial communities. Herein, this review examines challenges in DDS development, focusing on enhancing antibacterial activity and eradicating biofilms without adverse effects. Furthermore, advances in immune system modulation and photothermal therapy are discussed as future directions for the treatment of bacterial diseases.

## 1. Introduction

Bacterial pathogens pose various infections to human health worldwide through the development of multi-drug resistance [1]. For example, methicillin-resistant *Staphylococcus aureus* (MRSA) is listed as a multi-drug-resistant pathogen by the World Health Organization (WHO) and causes various infections in humans. Bacterial diseases are resistant to antimicrobial regimens, including osteomyelitis, pneumonia, infective endocarditis, bacteremia, and implant-associated infections [2,3]. Moreover, bacterial pathogens develop biofilm matrices by generating extracellular polysaccharides (EPS). The EPS facilitates cellular communication between the bacterial cells via quorum sensing (QS), which prevents nutrient competence and enhances the ability to respond to various environmental factors [4,5]. The EPS matrix acts as a bio-shied for bacterial pathogens, protecting them from immune response and various antimicrobial treatments [6]. Biofilm-forming bacterial infections are more hazardous compared to planktonic bacterial infections. The biofilm of the bacterial pathogen is 1000-fold more resistant to antibacterial treatments than planktonic bacterial cells of the same strain [1]. The global view towards antimicrobial resistance is one of angst, with bacteria evolving novel ways to evade drug-microbe interactions. Some current evasion mechanisms include efflux pumping, modification, inactivation, and limiting targeted drug uptake [7]. Therefore, an effective biofilm eradication approach is essential for treating biofilm-associated bacterial infections.

Nanotechnology and nanomedicine provide stimuli-responsive DDSs for triggering bacteriostatic and bactericidal effects in biofilm-forming bacterial pathogens. This method allows for greater drug efficacy, controlled localized drug delivery, carrier leakage prevention, and less adverse effects. Several studies have utilized a polymer modifications approach to increase specificity and drug efficacy in biomedical applications [8,9,10]. For instance, curcumin (Cur) and indocyanine green (ICG) co-encapsulated into the zeolitic imidazolate framework-8 (ZIF-8)/polylactic acid (PLA) coated with phase-change material (PCM) (Cur-ICG@ZIF-8/PLA/PCM) nanocomposite has been observed to have photothermal and photodynamic activities, enhancing bactericidal effects by 99% against MRSA and *E. coli* [11]. In addition, Cur-ICG@ZIF-8/PLA/PCM scaffolds effectively promote MRSA-infected wound healing under near-infrared (NIR) irradiation [11]. However, the efficiency of DDS is impacted by common exogenous and endogenous stimuli, including enzymatic activity, H_2_O_2_, pH, temperature, ions, electrical, light, magnetic fields, and ultrasound. The administration route also impacts the efficacy of the delivered drug. These routes can be enteral or parenteral, each possessing limitations with drug carrier types (hydrogel, polymer dot, nanoparticles, liposomes) [12]. 

The most significant smart system increases efficacy due to higher local concentrations, reduced systemic side effects, and the released agent’s capacity to diffuse into the peri-implant tissues, killing bacteria on implant surfaces and in the surrounding environment [13]. Another work demonstrated that a hydrophilic and viscous hydrogel composed of titanium (Ti), red phosphorus (RP), poly (vinyl alcohol) (PVA)/chitosan (CS) hydrogel (PCP), and NO donor of S-nitro succinic acid (RSNO) (Ti-RP/PCP/RSNO) system releases NO to trigger the osteogenesis and MRSA biofilm eradication under NIR irradiation through immunotherapy and phototherapy [14]. The multi-stimuli (pH and electro responsiveness) chitosan-graft-polyaniline (CP) and oxidized dextran (OD) (CP/OD) hybrid hydrogels enhanced antibacterial activity against *E. coli* and *S. aureus* while showing excellent cytocompatibility, in vivo biodegradability, and biocompatibility [15]. The metal organic framework (MOF) DDS of ZIF-8 (zeolitic imidazolate framework 8) is a promising drug delivery nanocarrier with a porous structure. For example, dual-stimuli (NIR/pH)-responsive release of vancomycin’s (Van) from Van@ZIF8@PDA (van- ZIF-8 with polydopamine (PDA)) at pH 4.7 with NIR irradiation showed the enhanced in vivo antibacterial activity [16]. Certain drug encapsulation nanoparticles (NPs) can also be toxic, carcinogenic, and non-metabolizable to the body; thus, biodegradable NPs have been developed [17,18]. Stimuli pathology or physiology that aids in drug release is another factor to consider. Biofilm formation from bacterial infection poses a significant barrier and must be considered in DDS designs [19]. Thus, modeling proper stimuli-response DDSs and stimuli intensity is crucial. This review discusses in-depth roles and challenges of stimuli-response DDSs in the treatment of bacterial diseases.

## 2. Drug Antimicrobial Mechanisms and Encapsulation

### 2.1. Drug Carrier Types 

Various drug carriers used as vehicles include NPs, microbots, liposomes, dendrimers, and micelles that release the drug to localized sites. NPs have garnered considerable attention as they can deliver antimicrobial components directly to the infection site. Microbial growth in wounds hinders the healing process and often results in infection. Although antimicrobial creams are widely used, they offer inaccurate healing compound doses. Therefore, wound dressings are preferred, as they prevent secondary infections. Silver sulfadiazine (SSD) is an antibacterial drug that combats infections and prevents sepsis. However, due to its poor aqueous solubility and inability to bond to textile fibers, El-Feky et al. [20] observed the controlled release of SSD from chitosan NP wound dressing. Chitosan NPs, as a drug carrier and effective fabric coating material, have been studied through ionotropic gelation, where the positive charge of the chitosan amino group interacts with the negative charge of the polyanion. This wound dressing, coated with SSD-loaded chitosan NPs, exhibited an extended SSD delivery of over 24 h, inhibiting Gram-positive and Gram-negative bacterial pathogens. Reactive oxygen species (ROS)-responsive mesoporous silica nanoparticles (MSNs) composed of vancomycin (Van) thioketal (TK)-functionalized methoxy poly (ethylene glycol) (mPEG-TK) (Van-mPEG-TK-MSNs showed excellent antibacterial activity and *S. aureus*-infected wound healing activity [21]. Microrobot and liposome biohybrids are used for targeted delivery of drugs into infected sites with minimal invasion. For instance, microrobots loaded with photothermal agents and chemotherapeutic molecules, composed of magnetic and nanoliposomes sensitive to pH or external stimuli such as light and magnetic fields, are designed to deliver drugs to a specified site for killing *E. coli* [22]. Moreover, PEGylated liposomes are used for cancer therapeutics [23]. Simonis et al. have reported the efficiency of cationic liposomes in penetrating the blood–brain barriers (BBBs) [24]. Dendrimers are highly branched macromolecules with entrapment properties useful in DDS; among dendrimers, polyamidoamine (PAMAM) dendrimers are widely studied. These macromolecules possess vacant cavities to entrap cargo molecules and many functional groups that enhance their high solubility, making them reliable drug carriers [25]. Sulfonamides have a broad antibacterial activity spectrum but a considerable drawback of extremely low solubility in aqueous solutions. For example, Ma et al. researched sulfamethoxazole (SMZ) encapsulation into PAMAM dendrimers, revealing prolonged drug release and increased antibacterial activity against *E. coli* [25]. In addition to these materials, nanotechnology offers several innovative DDSs that precisely target specific bacterial infections, enhancing treatment efficacy and minimizing side effects. These are summarized in Table 1.

### 2.2. Targeted Stimuli for Bacterial Therapy

In addition to nanocarriers, targeted stimuli in bacterial therapy are gaining immense attention. Developing new methods for controlled drug release is crucial for maintaining efficient drug circulation for a prolonged period and reducing side effects [52]. Hence, stimuli-responsive drug release in localized infection sites is a promising avenue. Drug carriers are modified to deliver drugs when exposed to specific stimuli. ROS is a redox stimulus that includes hydrogen peroxide, hypochlorite, superoxide, and hydroxyl radicals. ROS is often found in living systems as an integral component of vital biochemical pathways [21]. Andoy et al. used polydopamine NPs (PdNPs) as a biocompatible photothermal agent and assessed their applicability in bacterial therapy against drug-resistant *E. coli* [26]. Li et al. [21] developed vancomycin-loaded thioketal-functionalized methoxy poly (ethylene glycol) (mPEG-TK) mesoporous silica nanoparticles (MSNs) sensitive to ROS for improved antibacterial drug delivery to kill *S. aureus.* In addition to the external stimuli, several studies explored endogenous stimuli-driven antimicrobial release for bacterial therapy. 

### 2.3. Mechanisms of Bacterial Therapy

Antimicrobials are one of the most prevalent treatments in antibacterial therapy. However, due to their constant misuse and overuse, humans now confront antimicrobial-resistant bacterial species. Introducing PTT-mediated bacterial therapy techniques is critical in overcoming this challenge [16]. Antibacterial activity involves various biocidal mechanisms including membrane lytic activity, enzyme inactivation, and ROS induction [26]. The bacterial cell’s mechanical integrity is tested during bacterial therapy. Disrupting the elasticity of the cell membrane or envelope enhances drug entry into the bacterial cell [26]. Drug carriers that respond to certain stimuli combine antibacterial mechanisms to kill or inhibit bacteria. Antimicrobial peptides (AMPs) outshine conventional antimicrobials in hindering antimicrobial resistance [26]. 

Laser-induced antimicrobial functionality has significantly lowered the critical temperature required to inactivate bacteria in the infection site. This antibacterial system eradicated *E. coli* cells by transferring heat directly to their envelope and inducing structural deterioration. In photothermal therapy, NIR light is converted into heat, which is then used to kill microbes. For example, Wang et al. [60] designed a ZIF-8-based antibacterial system capsulated with a Pd-Cu nanoalloy as the photothermal agent and the antimicrobial amoxicillin. This system incorporates two bacterial film eradication mechanisms: chemotherapy and photothermal therapy. The chemotherapy mechanism releases amoxicillin in response to pH changes, especially in acidic environments. The release disrupts the bacterial wall, notably affecting planktonic bacteria (G+/G−) and their biofilms (*S. aureus* and *P. aeruginosa*), and the release is sped up by the co-released Pd-Cu nanoalloy. This photothermal agent converts NIR irradiation light energy into heat, synergizing with amoxicillin to kill the bacteria [60]. 

**Figure 1:**
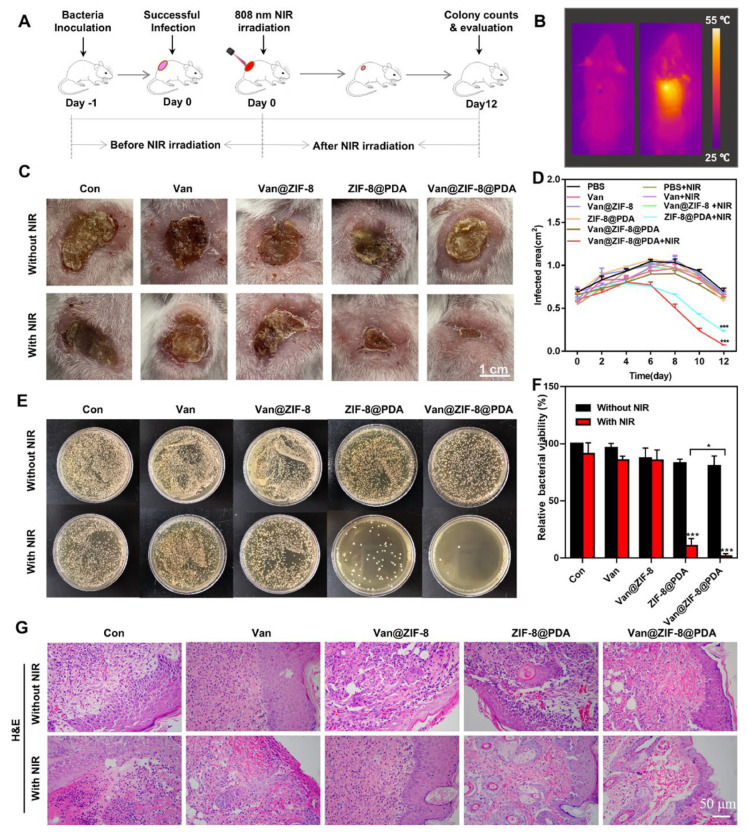
The NIR irradiation process for treating skin infection in mice (**A**–**G**). Data are expressed as means ± s.d., n = 3, * *p* < 0.05, *** *p* < 0.001. Reprinted from Xiao et al., [16] Acta Biomaterialia (122, 2021) with permission from Elsevier (License Number: 5806490081467).

Xiao et al. [16] developed a zeolitic imidazole framework-8 (ZIF-8) modified with polydopamine, with vancomycin (Van) encapsulated to form a Van@ZIF-8@PDA formulation. This material releases vancomycin in response to pH changes and hypothermia from NIR irradiation. After 10 min of NIR irradiation, the drug release percentage was 65% at a 4.7 pH and 38.7% at a 7.4. These results suggested that the antibacterial system damaged the bacteria’s genomic DNA through NIR irradiation. PDA was added as a ZIF-8 surface modification and implemented photothermal activation. This dual-stimuli-responsive antibacterial system expressed synergistic germicidal and antibiofilm properties, proving remarkable as a drug-resistant bacterial treatment. This system was also proven effective in a mouse model, evidence of the healing of *E. coli*-infected wounds (Figure 1).

**Figure 2:**
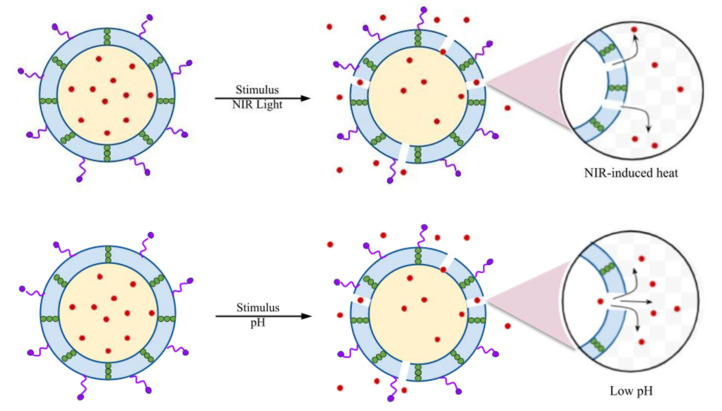
NIR- and pH-responsive drug release profile through membrane permeabilization. Modified from [22]. The red color dots—Doxorubicin (DOX), arrows indicate the stimulus responsive release of drug. Distributed under a Creative Commons Attribution License 4.0 (CC BY).

Wu et al. developed a liposome-based nanoreactor that releases drugs in response to the expression of bacterial toxins as an endogenous stimulus [52]. When the liposome-based nanoreactor is exposed to MRSA, which produces these bacterial toxins, the toxins penetrate the nanoreactors and form pores while maintaining the reactor structure. Water enters the membrane through these pores, reacts with CaO_2_, and produces H_2_O_2_. The produced H_2_O_2_ decomposes into O_2_, aiding in the release of antimicrobial or any antibacterial material through significant reactor expansion and reducing toxin toxicity. Therefore, this system is applicable for therapeutic use against bacteria that secrete pore-forming toxins. In another study, Akolpoglu et al. [22] demonstrated that a liposome-based bacterial microbot DDS releases its cargo in response to a stimulus such as NIR and pH. A fluorescent dye capable of absorbing NIR light was inserted into the lipid membrane. The absorbed NIR light was converted into heat, causing structural modification in the membrane to release the cargo. The lipid bilayer of the liposome-based carrier undergoes membrane permeabilization by transitioning from the gel phase to the liquid crystalline phase (Figure 2).

Furthermore, drug release profiles were investigated over ten days with pH levels ranging from 2.5 to 7.4. Results revealed that drug release was higher at lower pH, with 98% released within six days at pH 2.5. The protonation of the carboxyl group in the membrane facilitated this release by disrupting the liposomal membrane at lower pH [22]. Li et al. developed a surface-modified ROS-responsive MSN-based antimicrobial delivery system that encapsulated vancomycin to heal *S. aureus*-infected wounds [21]. As the concentration of H_2_O_2_ in the medium increased, the vancomycin release rate also increased due to the disintegration of mPEG-TK MSN. Assays revealed that this mPEG-TK-MSN system used to treat *S. aureus* infection partially disintegrated the bacteria’s cell wall or membrane, allowing to pass through a fluorescent dye [21]. Additionally, carbon quantum dots (CQDs) are used in drug cargo due to their unique biocompatibility. Huang et al. [33] incorporated CQDs into polylactic-co-glycolic acid (PLGA)-based NPs, which had previously been established in bacterial biofilm treatments. The synthesized CQD-PLGA indicated efficient loading of antimicrobial drugs, such as azithromycin and tobramycin, and released the drug based on laser irradiation. The CQDs rapidly converted NIR to heat, disrupting the PLGA nanomembrane network, increasing bacterial membrane permeability, and releasing the drug. Thus, the azithromycin-loaded CQD-PLGA system demonstrated antibiofilm properties against *P. aeruginosa* [33]. 

## 3. Controlled Antibacterial Drug Delivery Development 

In recent years, many ground-breaking strategies for creating DDSs have been devised and are currently in use. An essential feature of an effective DDS is its ability to deliver proper drug concentrations to target areas, and thereby enhance medication bioavailability. Furthermore, selecting a suitable delivery carrier is a significant challenge. Because of their biocompatibility and ease of production, liposomes, microspheres, nanomaterials, polymeric particles, and other drug carriers are widely employed today [61]. As a result of their precise target delivery and programable drug release mechanisms, the demand for these treatments to combat increased antimicrobial resistance and microbial infection has dramatically risen.

### 3.1. Polymer-Based Exosome Modification 

Hydrogels comprising natural or synthetic polymers are widely used for injections and topical therapeutics due to their self-supporting and 3D viscoelastic networks. Although they provide excellent applications, their lower tensile strength impacts drug loading distribution. A study on a biocompatible and degradable dual-delivery nanogel system, synthesized by allyl-functional hyperbranched dendritic-linear-dendritic copolymers and fabricated via thiol-ene chemistry, demonstrated that hydrogels are primarily used as wound healing treatments [62]. These hydrophobic antimicrobial ciprofloxacin nanogels exhibited a 2.83 wt% drug loading capacity, enabling a prolonged antimicrobial release and significantly reducing bacteria (*S. aureus* and *E. coli*) in vitro [62]. 

**Figure 3:**
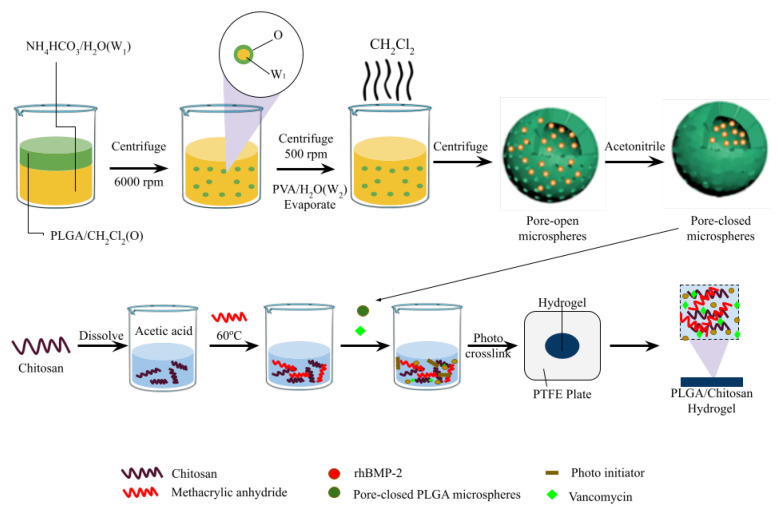
CM/VAN/MPs hydrogel film fabrication. Modified from [63] with permission from Elsevier. This figure was created with biorender.com; License number: 5806481251498.

Hydrogels are currently produced with more than one attribute. For instance, a study developed an antibacterial and osteogenic hydrogel loaded with vancomycin and recombinant human bone morphogenetic protein-2 (Figure 3). Poly (lactic-co-glycolic acid) is extensively used to develop controlled DDSs due to its enhanced biocompatibility, high encapsulation efficiencies, and biodegradability. Additionally, to avoid the cellular damaging effects of conventional cross-linking agents, the photo-crosslinking method was employed, which allowed for programable reaction time and rapid gel formation [63]. This DDS reportedly shows an excellent antibacterial effect against *S. aureus* in both in vitro and in vivo model experiments [63].

**Figure 4:**
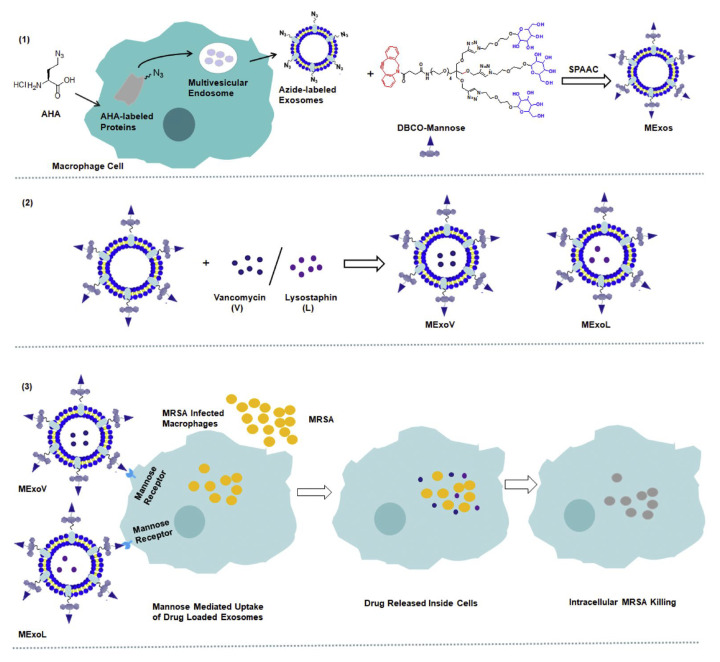
MExoV (vancomycin)- or MExoL (lysostaphin)-loaded mannosylated exosome fabrication. Reprinted from [65] with permission from Elsevier (License Number: 5806490380839).

Exosomes are a promising DDS due to their superior delivery efficiency, biocompatibility, and lower immunogenicity. These vesicles, enclosed by a 40–200 nm membrane, form via the fusion of multivesicular bodies with cell plasma membranes. For instance, Yang et al. [64] conveyed that exosomes could serve as excellent DDS carriers for antimicrobial therapy, capable of loading both hydrophilic and lipophilic drugs due to their lipid bilayer. Similarly, a mannose-modified exosome DDS was designed to deliver lysostaphin and vancomycin in a nanocomposite platform to bacterial infection sites to eradicate MRSA [65]. In this method, azides were incorporated into exosomes by attaching DBCO-mannosyl ligands to azide-integrated exosomes, altering the metabolic function of exosome-secreting cells [65] (Figure 4). 

Another study demonstrated that selective laser melting can fabricate 3D porous bio-ceramic (Si–CaSiO_3_) scaffolds with an even, spherical macropore structure, approximately 400 μm pore size, and 35% porosity. In addition, mesopores were obtained with pore sizes ranging from 15 to 50 μm. The controllable porosity at both macro- and meso-levels combined with a biocompatible polymer (PCL) coating allows for scaffold production aimed at bone regeneration and sustained vancomycin release [66]. Furthermore, the Plackett–Burman factorial design has been utilized to create calcium alginate microspheres (Ca-SA) fortified with chitosan and dual antimicrobials encapsulated in chitosan-based-Ca-SA. The CS-Ca-SA microspheres exhibited a surface pH of 6.5 ± 0.5 with enhanced muco-adherence and reduced swelling and erosion compared to Ca-SA microspheres. This system showed significant antibacterial action against *S. aureus* and *E. coli* and was cytocompatibility with L929 cell lines [67]. This study confirmed that dual polymer and drug-based microspheres are biodegradable, stable, non-toxic, mucoadhesive, and capable of controlled drug release [67]. The solvothermal technique has successfully fabricated the Carboxymethylcellulose/MOF-5/GO bio-nanocomposite (CMC/MOF-5/GO). The GO and CMC/MOF-5/GO were encapsulated with tetracycline (TC), aiding in stomach pH regulation. This aspect was essential for TC release in the gastrointestinal tract, ensuring the long-term stability of dosage-dependent drug release. Antibacterial activity against *E. coli* was enhanced as compared to non-MOF-loaded TC trials [61].

### 3.2. Inorganic Nanomaterials-Based Modifications 

**Figure 5:**
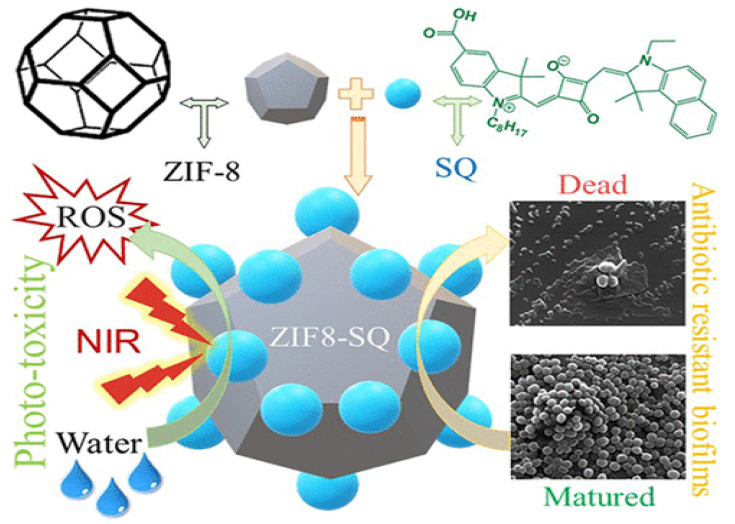
ZIF-8 encapsulated squaraine photo-toxicity and antimicrobial resistivity. Reprinted from [70] with permission from Copyright © 2019, American Chemical Society.

Metal-organic frameworks (MOFs) have found successful applications in DDSs due to their high porosity, programmable composition, structure manipulation, large surface area, intrinsic biodegradability, functionality, and biocompatibility [61,68,69]. Their ability to load drugs efficiently and prevent drug leakage make them promising tools for drug delivery. ZIF-8, a subclass of MOFs, is a porous crystalline material formed by zinc ions and 2-methylimidazole coordination. ZIFs exhibit pH-responsive degradability; for example, ZIF-8 degrades under acidic conditions while maintaining structural stability under normal physiological conditions [70,71]. This pH-sensitive property makes it an ideal nanocarrier for delivering therapeutic drugs. Bagchi et al. [70] investigated nano-MOFs that encapsulated squaraine (SQ) drugs for PDT to combat drug resistance in planktonic bacteria and biofilm formations. They reported on ZIF-8 MOF nanocrystals cohered to SQ (ZIF8-SQ), demonstrating a gravimetrically analyzed thermal stability up to 450 °C and a drug loading capacity of approximately 31%. ZIF-8 increased the drug loading capacity to 39.2%, indicating its complementary structuring effect. Upon exposure to 650 nm radiation, the nano-MOF exhibited pH-sensitive release of ROS and dual-stimulus responsiveness. This action effectively disrupted MRSA biofilms, causing functional and complete adherence loss to structurally robust bacterial biofilms (Figure 5) [70]. Nano-based DDSs can be programmed to respond to various stimuli types: endogenous, exogenous, or both. A demonstration of near-field IR and pH stimuli-response using ZIF-8 (MOF) with a surface PDA configuration and encapsulated vancomycin showed excellent antibacterial activity through photothermal degradation, membrane disruption, and cellular damage against planktonic Gram-positive and Gram-negative bacteria and their respective biofilms. This nanoparticle-based DDS exhibited superior biocompatibility, photothermal conversion, pH-triggered drug release, and NIR-mediated drug release, potentially enhancing therapeutic efficacy. Further in vivo, cytotoxic studies on a Mu50 mouse model with skin abscess confirmed the effectiveness and non-toxicity of the NP-based DDS [16]. Several studies have provided insights into targeted drug delivery using MOF-based DDSs at specific pathological sites, summarized in Table 2.

## 4. Combination Therapy

Combinational therapy allows for dual or multi-therapeutic delivery to targeted sites. For example, ZIF MOFs are promising gatekeepers as they respond to UV and pH stimuli. This light-initiated sequential reaction involves a jump reagent for pH activation triggered by UV radiation, which generates acid for MOF degradation, thereby releasing antimicrobial and zinc in a dose-dependent and controlled manner [71]. This targeted delivery demonstrates synergistic actions such as preventing wound infections and enhancing wound healing [71]. Ciprofloxacin (CIP)-loaded ZIF-8 (CIP-ZIF-8), which is pH-dependent, demonstrated a 21 wt% drug-loading capacity. The drug release rate was slower at pH 7.4 than at mildly acidic conditions (pH 5.0). Their combined activity against Gram-positive and Gram-negative species indicated enhanced microbial growth inhibition compared to the control [97]. Another MOF-53(Fe)@Vancomycin DDS had a 20 wt% drug-loading capacity. The MOF-53(Fe) exhibited the highest degradation percentage of 0.75% at pH 7.4 and 0.17% at pH 5.5, which is essential for a DDS. This study suggests that if a bacterial infection induces antimicrobial release, MOF-based carrier systems are applicable for surgical implants in acidic environments, establishing 99.3% antibacterial efficacy and non-adverse drug release therapy [68]. Synergistic DDSs provide an edge over traditional DDS approaches, overcoming drug-resistance development by bacteria and ensuring efficient bacteriostatic or bactericidal effects. A study that used water phase self-assembly of tetracycline (Tet)@ZIF-8@ hyaluronic acid (TZH) demonstrated a triple effect system: targeted pH-dependent drug release from the MOF cage, a synergistic antibacterial effect of zinc ions being released, and TZH triggering the hyaluronic acid-mediated pathway in CD44R cells. These effects indicated a clearance rate of over 98% [69].

## 5. Conclusions and Future Prospects 

The rise of antimicrobial-resistant bacterial species has underscored the limitations of conventional methods, which often exacerbate antimicrobial resistance rather than suppression. Therefore, there is an urgent need for novel, effective, and safe DDSs for developmental therapeutics for bacterial diseases. Various mechanisms have been developed to combat bacterial infection, ranging from biohybrid systems and mediated bacterial therapies to multiple stimuli-responsive DDSs. These innovative methodologies surpass conventional therapeutic approaches by offering higher bioavailability, targeted treatment, drug delivery, and reduced antimicrobial leakage, ultimately providing safety by means of non-cytotoxicity. 

Despite the development of several inorganic and organic DDSs for eradicating bacterial biofilms and treating infections, most of the studies have been confined to laboratory and in vivo mice experiments. The transition from these experimental stages to commercial products in medical or industrial settings remains limited. This review has explored various drug carrier types and their bacterial therapy mechanisms through mediated or target-specific stimuli. Stimuli-based DDSs have the potential to resolve problems faced by conventional DDSs, but they are not without drawbacks. One limitation is accessibility, as these methods are relatively expensive. Another challenge is the variability in individual physiochemical compositions and reactions, leading to fluctuations in efficacy. In certain scenarios, the stimulus may be less intense or vice versa, leading to dosage imbalances. This review substantiates that these DDSs improve efficacy, site targeting, and biocompatibility. However, further investigation is still necessary to enhance the practical application of these systems.

To tackle the challenges faced by current DDSs, future research must focus on several pivotal areas. Developing cost-effective methods for synthesizing and implementing stimuli-responsive DDSs is essential to make these advanced treatments more accessible and affordable in clinical practices. Additionally, advancing personalized DDSs that consider individual physiological variations will ensure consistent and effective treatment outcomes across diverse patient groups. The transition from laboratory research to commercial products could be facilitated by conducting extensive clinical trials and forging partnerships with pharmaceutical companies. Enhancing the sensitivity of DDSs to stimuli is another critical area, as it will ensure precise dosage control and minimize the risks associated with incorrect dosages. Establishing comprehensive regulatory guidelines might help streamline the approval process for new DDSs, ensuring they are both safe and effective while speeding up the availability of them on the market. Furthermore, incorporating sustainable materials and eco-friendly fabrication processes into the development of DDSs might reduce their environmental impact. Addressing these areas will significantly advance the adoption of innovative DDSs, leading to more effective and safe treatments for bacterial diseases and helping to combat the growing issue of antimicrobial resistance.

## Figures and Tables

**Table 1:** Summary of stimuli-responsive antibacterial systems. phosphorylation-modified poly (ethylene glycol)-b-poly(tyrosine) (PEG-b-PPTyr). amphiphilic fluorinated copolymers were assembled into micelles (FCBMs); ciprofloxacin (CIP); curcumin (Cur); chitosan (CS); silver nanoparticles (AgNPs); cinnamaldehyde (CA); chitosan nanoparticles (CSNPs); solid lipid nanoparticles (SLNs); polydopamine (PDA); 2-(dimethylamino)ethyl methacrylate (DMAEMA); butyl methacrylate (BMA); and 2-propylacrylic acid (PAA); carboxybetaine-co-dopamine methacrylamide (PCBDA); contact lenses (CLs); xanthan gum (XG); aloe vera extract (AVE); silica oxide nanoparticles (SiO NPs); ampicillin (Amp); erythromycin (Ery); 2,3-dimethyl maleic anhydride (DA); lysine and arginine and encapsulating ursolic and oleanolic acids (UOACDs); platensimycin (PTM).

Nanomaterial	Name of DDS System	Drug	Targeted Bacterial Infections	Mechanism of Action	Ref.
Nanoparticles	mPEG-TK-MSN	Vancomycin	*S. aureus* infected wound healing therapy	Cell membrane/cell wall partial disintegration	[21]
	Antimicrobial peptide-Polydopamine nanoparticles (PdNPs-AMP)	Antimicrobial peptides (AMP)	*E. coli*	Structural deterioration	[26]
	CS-AgNPs	Silver nanoparticles	*E. coli* and *S. aureus*	-	[27]
	CSNP-CAs	Cinnamaldehyde	*S. aureus* biofilms eradication	Cell wall damage and permeability	[28]
	Rifampin-SLN-P-SA6	Rifampin	*S. epidermidis* biofilm eradication	-	[29]
	Cefazolin-containing niosome nanoparticles	Cefazolin	MRSA	Biofilm removal	[30]
	Ferromagnetic Nanoparticles (Fe_3_O_4_@PDA@Mino)	Minocycline (Mino)	Periodontal biofilm eradication	Regulation of inflammatory response	[31]
	p(DMAEMA-co-BMA-co-PAA)	Farnesol	Treatment of rodent dental caries (*S. mutans* biofilms)	Bacterial biofilm penetration	[32]
	Quantum dots-Poly lactic-co-glycolic acid (PLGA) (CQD-PLGA NPs)	Azithromycin and tobramycin	Eradication of *P. aeruginosa* biofilms	Increases bacterial membrane permeability	[33]
	PCBDA@AgNPs-CL	AgNPs	Microbe-induced ocular infections (*C. albicans*)	Resist–kill–remove	[34]
	Pt-Se NPs	-	*S. enterica*, *E. coli*, *L. monocytogenes*, *S. aureus*, and *B. cereus*	Bacterial cell damage	[35]
	XG-AVE-Ag/MgO NCs	Ag and MgO, nanoparticles, Aloe vera extracts	*E. coli biofilm* removal	Cell wall damage	[36]
	Pae-SiO_2_ NPs	Paeoniflorin	*S. aureus* and *B. cereus*	-	[37]
	ZrO_2_-Amp NPs and ZrO2-Ery NPs	Ampicillin and erythromycin	*E. coli* and *B. cereus*, in vitro wound healing	Protein and DNA damage	[38]
	Tetracycline-loaded ZrO_2_ NPs	Tetracycline	*S. entrica* and *S. aureus* biofilm eradication	Penetration inside the biofilm	[39]
	CS-FeNPs	Fe NPs	*E. coli* biofilms eradication	Protein leakage, cell wall permeability	[40]
	Anti-CD54@Cur-DA NPs	Curcumin	Treatment of chronic lung infection (*P. aeruginosa*)	Inhibiting efflux pump-related genes	[41]
Micelles	Nanostructured antimicrobial micelles (CT9W1000 micelles)	Antimicrobial peptides (T9W)	*P. aeruginosa* lung infection	ROS production and anti-inflammatory effect	[42]
	SIR-micelles conjugated mannose targeting ligands	Inflammatory cytokines	Treatment of pneumonia infection of multidrug-resistant *K. pneumoniae*	Regulate the inflammatory cytokines	[43]
	Curcumin-loaded polymeric micelles	Curcumin	*S. aureus*, *E. coli* and *C. albicans*	-	[44]
	Caffeic acid graft chitosan copolymer loaded QR micelles (CA-g-CS/QR)	Quercetin	*E. coli*	In vivo antibacterial activity in broiler chickens	[45]
	Polyzwitterionic micelles	Triclosan	*S. aureus* infection	Drug penetration inside the biofilm kills bacteria	[46]
	Chitosan oligosaccharide lactate (COL)-pluronic F127 polymers, loaded with gatifloxacin (Gati@FCOL1/Gati@FCOL2 micelles)	Gatifloxacin	Eradication of *P. aeruginosa* and *S. aureus* and treatment of bacterial keratitis	Anti-quorum sensing (QS) effect	[47]
	Antibacterial Micelles- Carboxymethyl Chitosan (CC)/Oxidized Konjac Glucomannan (OKG) stevioside-stabilized honokiol (HS) (CC45/OKG40/HS hydrogel)	Honokiol	*S. aureus* infected Wound Healing	Eradicate the bacterial infection and regulate the inflammatory response	[48]
	PEG-b-PPTyr micelles	α-helical cationic polypeptide	*E. coli* infected wound healing	Eradication of bacterial biofilm and regulating the anti-inflammatory response	[49]
	CIP@FCBMs	Ciprofloxacin	Eradication of biofilms and MRSA-infected wound healing	Targeting the bacterial proteins and nucleic acid synthesis	[50]
	Cur-EPS conjugate-based polymeric micelles	Curcumin	Antioxidant, eradication of *E. coli*, *S. aureus*, *P. aeruginosa*, *S. typhimurium*, and *S. marcescens* biofilms	Antibacterial, antibiofilm, and antioxidant mechanisms	[51]
Liposomes	Liposome-based nanoreactor (RFP-CaO_2_@PCM@Lec)	Eutectic antimicrobial mixture	Treatment of MRSA-infected wounds	Antimicrobial release through pore formation	[52]
	Liposome-based bacterial microbats	Liposomal drug	*E. coli* infection	Lipid bilayer permeabilization	[22]
	Asiaticoside-Loaded Liposomes (rColMA/QCSG/LIP@AS/Ag@MOF (RQLAg) hydrogel	Asiaticoside	*E. coli* and *S. aureus*	Destroy the cell membrane	[53]
Dendrimers	Amino acid-conjugated cationic dendrimers (CDs)	UOACDs	*E. coli*, *K. pneumoniae*, MRSA, and MRSE	-	[54]
	PLGA/PTM; PAMAM/PTM NPs	PTM	*S. aureus* (mouse peritonitis model)	*S. aureus* cell membranes interactions	[55]
	Ag-loaded poly(amide-amine) dendrimer	Ag	*E. coli* and *S. aureus*	-	[56]
	Dendrimer G4 poloxamer nanoparticles	Coumarin	MRSA	Drug penetration and uptake, cellular damage	[57]
	Erythromycin-conjugated nano dendrimer	Erythromycin	*S. aureus*, *S. epidermidis*, *S. saprophyticus*, and *P. aeruginosa*	Membrane permeability and bacterial lysis	[58]
	Gelatin and gelatin Star-shaped polyamidoamine (PAMAM) dendrimer G3.5 (sIPN NCs)	Silver acetate	*S. aureus* and *P. aeruginosa*	Release kill mechanism	[59]

**Table 2:** Metal–organic framework (MOF) drug delivery systems’ stimuli response for enhanced antibacterial activity. Metal–organic framework (MOF); doxorubicin (DOX); tragacanth gum-g-poly (NIPA-co-VOE)-cl-poly(MBA) hydrogels (TGIAVE); mesoporous zinc-imidazolate derivative MOF (mesoMOF); cisplatin (cis-Pt); konjac glucomannan (KGM); glucose oxidase (GOx); norfloxacin (NOR); polyvinyl alcohol (PVA); lignin (Lig); vancomycin (Van); ZIF-8-derived porous carbon (ZDPC); 5-Fluorouracil (5FU).

Composite\Carrier Composition	MOF Average Size	Drug	Bioactivity	Drug Loading Capacity	Reference
van@ZIF-8@PDA	175.9 ± 2.74 nm	Vancomycin	Eradication of *S. aureus* biofilms and treatment of bacteria-infected wounds	6.71%	[16]
Pd-Cu nanoalloy ZIF-8	155.3 nm	Amoxicillin	Eradication of *P. aeruginosa* and *S. aureus* biofilm; *S. aureus* infected wound healing	-	[60]
ZJU-101	300 nm	Diclofenac sodium	-	(∼0.546 g/g)	[72]
UiO-66	1.22 nm pore diameter	Ciprofloxacin	24 mm (*E. coli*) 22 mm (*S. aureus)* inhibition zones	84%.	[73]
UiO-66-NH2	200 nm	Quinazoline	0.25–0.7 mg m/L MIC 0.25–4 mg m/L MBC		[74]
MIL-101(Cr)	SBET—10^3^ (m^2^ g^−1^ Vp—2.50 (cm^3^ g^−1^)	Ibuprofen and nimesulide	-	IBU, NMS (850, 443 mg g^−1^)	[75]
TGIAVE-Ag	25 nm	5-FU	Inhibited *K. pneumonia*, *P. aeruginosa*, *E. coli*, and *S. aureus*	89.13 ± 1.4%	[76]
Rifampicin@ZIF-8	157.96 ± 1.07 nm	Rifampicin	Inhibited *S. aureus*		[77]
Fe_3_O_4_@PAA@ZIF-8	50–200 nm	Ciprofloxacin	Inhibited *E. coli* and *S. aureus*		[78]
Hydrogel (CMC/PNIPAM-co-PAM).	39.782–38.235 g/g	Tetracycline	>85% scavenging efficiency		[79]
NCQDs/Dox/HA	4–6 nm, 4.89 nm diameter	Doxorubicin	Inhibited *S. aureus*		[80]
KGM/MOF Hydrogels	-	Honokiol, caffeic acid, osthole, baicalein, palmatine, pterostilbene, quercetin, and luteolin	*S. aureus*	0.09 mg/mg–0.157 mg/mg	[81]
MEL-loaded MOF (MM)	<1 µM	Antimicrobial peptides	MRSA	-	[82]
MOF(Fe-Cu)/GOx-polyacrylamide (PAM) gel	280 nm	Fe-Cu	*E. coli* and *S. aureus*; infected wound healing by modulation of antibacterial and inflammatory	-	[83]
Ag NPs@ACM-1	370 to 700 nm	AgNPs	*E. coli* and *S. aureus*	-	[84]
Curcumin-Loaded Zn-MOF Hydrogel	-	Curcumin	*E. coli* and *S. aureus*	-	[85]
Ca–Sr–AMN–MOF		Ca, Sr	*E. coli*	-	[86]
NOR-Fe_3_O_4_@ZIF-8 nanoparticles	20 nm	Norfloxacin	*E. coli*	-	[87]
Zn-MOF(ZIF-8)-PVA-Gel	98.72 nm	Zn-MOF(ZIF-8)	Infected wound healing and antibacterial activity against *S. aureus*	-	[88]
Isoniazid-loaded Cu-based metal-organic frameworks	-	Isoniazid	Inhibition of *Mycobacterium tuberculosis* biofilm	10%	[89]
CoCu-ZIF and ZnCu-ZIF	-	G-quadruplex/hemin DNAzyme-aptamer functionalized	MRSA	-	[90]
ZnO@ZIF-8	1.29 ± 0.45 μm	ZnO	*S. aureus*, and *P. aeruginosa*	30.23%	[91]
Lig-Van-MOF	242.48 ± 12.20 nm	Vancomycin	*E. coli* and *S. aureus*	84.25 ± 2.50%	[92]
Zn_3_[Fe(CN)_6_]/g-C3N4	500 nm	zinc hexacyanoferrate	*E. coli* and *S. aureus* and wound healing effect	-	[93]
Cu-MOF/CS	Pore size: 11.56 μm	Cu	*E. coli*, *P. aeruginosa*, *S. aureus*, and *MRSA* and *P. aeruginosa* infected wound healing	-	[94]
Ag_SA_-ZDPC	40–50 nm	Single atom-dispersed silver	*S. aureus* and *E. coli*	-	[95]
SnFe_2_O_4_-PBA/Ce6@ZIF-8 (SBC@ZIF-8)	50–100 nm	3-aminobenzeneboronic acid (PBA) and dihydroporphyrin e6 (Ce6)	*MDR S. aureus* infected wound healing	-	[96]

## Data Availability

The original contributions presented in the study are included in the article, and further inquiries can be directed to the corresponding author.
